# The effects of oclacitinib treatment on antimicrobial usage in allergic dogs in primary practice: an Australia wide case-control study

**DOI:** 10.1186/s12917-022-03255-y

**Published:** 2022-04-27

**Authors:** Hester Rynhoud, Catriona Croton, Grace Henry, Erika Meler, Justine S. Gibson, Ricardo J. Soares Magalhaes

**Affiliations:** 1 UQ Spatial Epidemiology Laboratory, School of Veterinary Science, The University of Queensland, Veterinary Science Building, Level 2, Room 235, Gatton, Queensland 4343 Australia; 2 Faculty of Health, Engineering and Sciences, School of Sciences, University of Southern Queensland, Toowoomba, QLD Australia; 3 School of Veterinary Science, The University of Queensland, Gatton, Queensland 4343 Australia; 4 Children’s Health and Environment Program, UQ Child Health Research Centre, The University of Queensland, South Brisbane, Queensland 4101 Australia

**Keywords:** Oclacitinib, Canine allergic skin disease, Antimicrobial use, Case-control study, Australia

## Abstract

**Background:**

Canine allergic dermatitis is a common diagnosis in veterinary practices which can lead to secondary infections requiring treatment with antimicrobials. A previous study suggested that dogs treated with oclacitinib in an Australian referral hospital required fewer courses of antimicrobial therapy compared to dogs receiving other anti-pruritic treatments. This study aimed to quantify the effect of oclacitinib treatment on the use of antimicrobials and other therapies in general practice veterinary clinics across Australia. A retrospective case-controlled review of patient records was designed to investigate the number of courses of antimicrobials and other therapies in dogs that received oclacitinib (Apoquel®), compared with those who received an anti-pruritic treatment that was not oclacitinib.

**Results:**

The target population included canine patients with a presumptive diagnosis of allergic dermatitis presenting between 2008 and 2018 to general practices contributing to the VetCompass Australia database. Patient records of interest were identified using search terms relating to allergic dermatitis, resulting in over 700,000 observations. Multivariable logistic regression models were developed to determine whether cases were prescribed fewer antimicrobial courses than controls, after adjusting for the presence of concurrent skin infections or infectious agents in ears. Our results indicate that fewer antimicrobial courses were prescribed in the cases compared to the controls. After adjusting for the concurrent skin infections, there was a significant reduction in the use of cefovecin [OR:0.62(0.39–0.98), *P* = 0.043], chlorhexidine [OR:0.57(0.42–0.77), *P* < 0.001], neomycin [OR:0.4(0.28–0.56), *P* < 0.001] and amoxycillin clavulanic acid (AMC) [OR: 0.55(0.39–0.78), *P* = 0.001] in cases compared to controls.

**Conclusion:**

This study demonstrates a potential sparing effect of oclacitinib on the prescription of antimicrobials for the treatment of allergic skin diseases in dogs. This information may assist in the planning of treatment for canine allergic dermatitis, with consideration for antimicrobial stewardship.

**Supplementary Information:**

The online version contains supplementary material available at 10.1186/s12917-022-03255-y.

## Background

Canine allergic dermatitis is an inflammatory and pruritic skin condition or disease, which can include canine atopic dermatitis (CAD), canine adverse food reaction (CAFR) and flea allergic dermatitis (FAD). The first steps in the management of allergic dermatitis are the identification and avoidance of allergens that can trigger potential flares and ensuring there is adequate maintenance and care to preserve skin barrier function.

The condition can be complex with multiple therapies available. Currently topical and oral corticosteroids, cyclosporine and oclacitinib are most commonly used as they are all core treatment options recommended by International Committee on Allergic Disease of Animals for the management of chronic atopic dermatoses [1–3]. A newer biologic therapy for treatment of chronic atopic dermatitis, lokivetmab (Cytopoint®) has been reported to have a more pronounced effect on pruritis compared to cyclosporin [4]. Antihistamines and essential fatty acid supplementation are other treatments; these are less researched or not commonly recommended as primary treatments [5].

Secondary infections are a known complication of canine allergic dermatitis. The most common bacterial isolate cultured from skin infections in allergic dogs is *Staphylococcus pseudintermedius* and the most common fungal agent is *Malassezia pachydermatis* [6]*.* These secondary infections are often associated with poor control of the disease [7]. An unnecessarily heavy dependence on antimicrobials for infection management, with potential for development of resistance, does not align well with good antimicrobial stewardship (AMS). Veterinarians are encouraged to follow AMS guidelines when treating allergic dermatitis cases to improve efficacy and limit further development of resistance [8, 9].

Treatments that prevent infections and/or decrease inflammation are expected to reduce the need for use of antimicrobials in allergic dermatitis. Oclacitinib effectively treats the symptoms of allergic dermatitis by inhibiting the function of a variety of pro-inflammatory, pro-allergic and pruritogenic cytokines [10]. Specifically, oclacitinib selectively inhibits Janus kinase 1 (JAK1) to reduce inflammation and pruritis in canine patients at least 12 months of age [1]. A previous study investigating the effects of oclacitinib use on antimicrobial use in a University referral hospital in Australia, suggested dogs treated with oclacitinib had less antimicrobial use compared to dogs treated with other anti-pruritic drugs [11].

This work expands upon data from this unicentric study, by providing more representative data from multiple Australian general practices on oclacitinib treatment and antimicrobial usage patterns in oclacitinib-treated patients [11]. The specific objectives were to identify any differences in antimicrobial and anti-pruritic treatments between cases and controls, and within cases (before and after oclacitinib use).

## Results

**Fig. 1:**
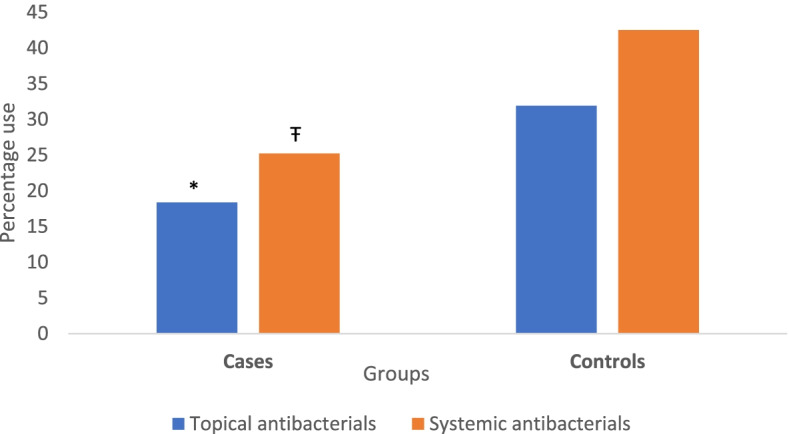
Systemic and topical antimicrobial use in 1345 cases and 5380 controls with allergic dermatitis for all consults included in the study. The sets of bars represent the percentages of topical and systemic antimicrobial courses prescribed in cases after oclacitinib use and controls. The symbols * and Ŧ represent a significant difference between the two groups (*P* < 0.05)

**Fig. 2:**
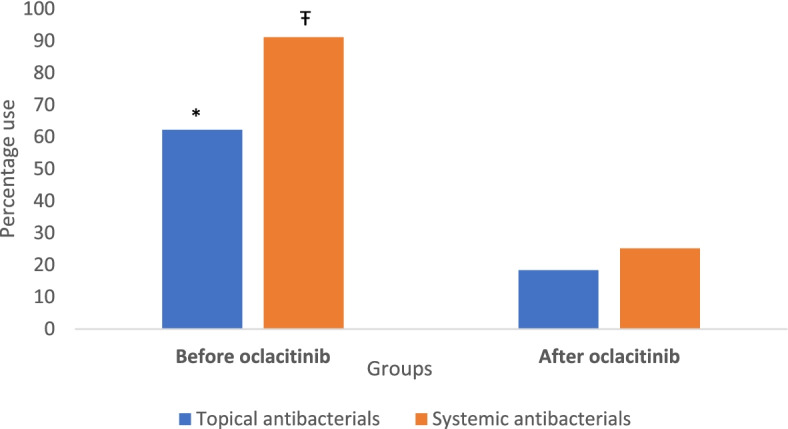
Systemic and topical antimicrobial use in 1345 cases with allergic dermatitis for all consults included in the study. The sets of bars represent the percentages of topical and systemic antimicrobial courses prescribed in cases before and after oclacitinib. The symbols * and Ŧ represent a significant difference between the two groups (*P* < 0.05)

The descriptive analysis revealed that there were significant differences in the percentage use of topical and systemic antimicrobials between cases and controls (*P* < 0.001), and before and after oclacitinib use (*P* < 0.001) (Figs. 1 and 2).

Majority of cases and controls were experiencing allergic dermatitis without infection at their initial consultation (first consultation with oclacitinib dispensation for cases), followed by superficial pyoderma (Supplementary Table 4). *Malassezia* was the most common agent present in ears with ear conditions in cases and controls, followed by cocci and then rods at their initial consultation.

### Univariable analysis

The univariable analysis revealed that age category, neuter status, breed size, total topical and systemic antimicrobial use and topical and systemic glucocorticoid use, allergy testing, and the use of medicated shampoo were significantly associated with the outcome of being a case (Table 1). Cases had significantly less odds of using cephalexin (OR: 0.76 [0.64–0.91]; *P* = 0.003), chlorhexidine (OR: 0.53 [0.39–0.72]; *P* < 0.001), neomycin (OR: 0.38 [0.27–0.53]; *P* < 0.001), and amoxicillin plus clavulanic acid (OR: 0.47 [0.34–0.66]; *P* < 0.001), compared to controls. The univariable analysis also suggested that pruritic dogs using oclacitinib had significantly higher odds of presenting with superficial pyoderma at their initial consultations compared to controls (OR: 1.41 [1.41–2.92]; *P* < 0.001).

**Table 1:** Univariable associations between Oclacitinib treated patients (cases) and patients treated with other therapies (controls)

Variable	Odds Ratio (95% Confidence Interval)	***P***-value*
Age		**< 0.001**
**1 to 4 years**	Reference	
> 4 to 8 years	0.88 (0.77–1)	0.053
> 8 years	0.58 (0.49–0.68)	< 0.001
**Sex**		
Male	Reference	0.676
Female	0.97 (0.85–1.10)	
**Neuter Status**		
Neutered	Reference	< 0.001
Entire	0.68 (0.56–0.82)	
**Breeds**		**< 0.001**
Small	Reference	
Medium	0.54 (0.44–0.65)	< 0.001
Large	0.5 (0.42–0.59)	< 0.001
**Total number of courses**		
All antimicrobials	0.73 (0.63–0.85)	< 0.001
All glucocorticoids	0.58 (0.48–0.72)	< 0.001
Topical antimicrobials	0.65 (0.52–0.81)	< 0.001
Systemic antimicrobials	0.69 (0.58–0.82)	< 0.001
Topical glucocorticoids	0.67 (0.54–0.84)	< 0.001
Systemic glucocorticoids	0.47 (0.37–0.6)	< 0.001
Antihistamines	0.39 (0.26–0.57)	< 0.001
**Total number of courses for individual drug**		
Cephalexin	0.76 (0.64–0.91)	0.003
Cefovecin	0.67 (0.43–1.04)	0.076
Chlorhexidine (2–5%)	0.53 (0.39–0.72)	< 0.001
Neomycin	0.38 (0.27–0.53)	< 0.001
Polymixin B	0.77 (0.57–1.04)	0.091
Amoxycillin plus clavulanic acid	0.47 (0.34–0.66)	< 0.001
**Other**		
Allergy testing (yes/no)	3.62 (1.18–11.11)	0.025
Hypoallergenic diet	1.25 (0.9–1.73)	0.175
Medicated shampoo	0.51 (0.38–0.68)	< 0.001
Flea treatments	0.81 (0.65–1.01)	0.067
**Evidence of concurrent skin infection at baseline**		
Non-infectious allergic dermatitis	Reference	**< 0.001**
No evidence of concurrent skin infection	0.62 (0.43–0.89)	0.008
Superficial pyoderma	1.41 (1.41–2.92)	< 0.001
Deep pyoderma	Omitted	
**Infectious agents in ear at baseline**		
No infectious agents present	Reference	**0.1043**
Cocci present	0.97 (0.63–1.50)	0.907
Rods present	0.40 (0.13–1.28)	0.124
*Malassezia* present	1.23 (0.97–1.56)	0.079

**P values are significant at the 0.05 level. The *P* values for the individual levels of the categorical variables compared to the baseline are given, with a bolded *P* value for all levels of that categorical variable combined

### Multivariable models

The multivariable model A (Table 2) revealed that after adjusting for age, sex, neuter status, breed, total drug groups use, and evidence of concurrent skin infections and the presence of infectious agents in ears at baseline, cases had lower odds of using topical (OR: 0.78 [0.62–0.98]; *P* = 0.034) and systemic (OR: 0.5 [0.39–0.64]; *P* < 0.001) glucocorticoids, antihistamines (OR: 0.48 [0.31–0.74]; *P* = 0.001) and medicated shampoo (OR: 0.69 [0.51–0.92]; *P* = 0.011) compared to controls. Cases also seem to have higher odds of getting tested for allergies (OR: 3.94 [1.27–12.18]; *P* = 0.017) and going on a hypoallergenic diet (OR: 1.77 [1.26–2.47]; *P* = 0.001). The odds of presenting with superficial pyoderma at the baseline consultation (OR: 2.71 [1.89–3.89]; *P* < 0.001) was significantly higher in cases compared to controls. There were higher odds of *Malassezia* identification in the ears of cases at baseline consultations compared to controls (OR:1.31[1.02–1.69]; *P* = 0.035).

**Table 2:** Multivariable model results displaying the association for drug groups (A) and individual drugs (B)

Model	A		B	
Variable	OR (95% CI)	***P***-value*	OR (95% CI)	***P***-value*
Age		**< 0.001**		**< 0.001**
**1 to 4 years**	Reference		Reference	
> 4 to 8 years	0.84 (0.72–0.97)	0.019	0.84 (0.73–0.96)	0.013
> 8 years	0.5 (0.42–0.6)	< 0.001	0.53 (0.45–0.63)	< 0.001
**Sex**				
Male	Reference		Reference	
Female	0.96 (0.83–1.1)	0.535	0.95 (0.83–1.09)	0.453
**Neuter Status**				
Neutered	Reference		Reference	
Entire	0.64 (0.52–0.78)	< 0.001	0.66 (0.54–0.81)	< 0.001
**Breeds**		**< 0.001**		**< 0.001**
Small	Reference		Reference	
Medium	0.53 (0.43–0.64)	< 0.001	0.53 (0.43–0.64)	< 0.001
Large	0.46 (0.39–0.55)	< 0.001	0.48 (0.4–0.56)	< 0.001
**Evidence of concurrent skin infection at baseline**		**< 0.001**		**< 0.001**
Non-infectious allergic dermatitis	Reference		Reference	
No evidence of concurrent skin infection	0.73 (0.5–1.06)	0.094	0.74 (0.52–1.06)	0.099
Superficial pyoderma	2.71 (1.89–3.89)	< 0.001	2.58 (1.81–3.68)	< 0.001
Deep pyoderma	Omitted		Omitted	
**Infectious agents in ear at baseline**		**0.073**		**0.16**
No infectious agents present	Reference		Reference	
Cocci present	0.99 (0.65–1.51)	0.965	0.91 (0.59–1.41)	0.687
Rods present	0.53 (0.18–1.55)	0.248	0.52 (0.17–1.56)	0.244
*Malassezia* present	1.31 (1.02–1.69)	0.035	1.23 (0.98–1.56)	0.076
**Total number of courses**				
Topical glucocorticoids	0.78 (0.62–0.98)	0.034		
Systemic glucocorticoids	0.5 (0.39–0.64)	0		
Antihistamines	0.48 (0.31–0.74)	0.001		
**Other**				
Allergy testing (yes/no)	3.94 (1.27–12.18)	0.017		
Hypoallergenic diet	1.77 (1.26–2.47)	0.001		
Medicated shampoo	0.69 (0.51–0.92)	0.011		
**Total number of courses for individual antimicrobial drug**				
Cefovecin			0.62 (0.39–0.98)	0.043
Chlorhexidine (2–5%)			0.57 (0.42–0.77)	< 0.001
Neomycin			0.4 (0.28–0.56)	< 0.001
Amoxycillin plus clavulanic acid			0.55 (0.39–0.78)	0.001

*P values are significant at the 0.05 level. The *P* values for the individual levels of the categorical variables compared to the baseline are given, with a bolded *P* value for all levels of that categorical variable combined

The multivariable models B (Table 2) revealed that after adjusting for age, sex, neuter status, breed, individual drug use, and skin and ear condition at baseline, cases had significantly lower odds of using cefovecin (OR: 0.62 [0.39–0.98]; *P* = 0.043), chlorhexidine (OR: 0.57 [0.42–0.77]; *P* < 0.001), neomycin (OR: 0.4 [0.28–0.56]; *P* < 0.001) and amoxicillin plus clavulanic acid (OR: 0.55 [0.39–0.78]; *P* = 0.001) compared to controls. Again, the odds of presenting at baseline with superficial pyoderma (OR: 2.58 [1.81–3.68]; *P* < 001) was significantly higher in cases compared to controls.

## Discussion

Antimicrobial stewardship in veterinary practice is an important intervention in the fight against antimicrobial resistance [9]. This includes the reduction in prescriptions of antimicrobials to pets whenever clinically possible. Allergic dogs often experience secondary infections, and an increase in the antimicrobial resistant infections over the past two decades emphasize the importance of AMS in veterinary dermatology [12]. The reduction of chronic skin inflammation can interrupt the itch cycle, restore a normal functioning skin barrier, and support resolution or prevent secondary infections [10]. Our previous study using data from a single referral hospital investigated whether the use of a specific anti-pruritic drug, oclacitinib, in allergic dogs would reduce the use of antimicrobials indirectly [11]. The results of this study suggested that pruritic dogs treated with oclacitinib did indeed experience a reduction in antimicrobial use compared to pruritic dogs treated with other therapies. Our present study aimed to build on these findings and test whether the magnitude and direction of the observed effect of oclacitinib on the number of antimicrobial courses and other treatment courses were similar in patients presenting to general practices across Australia.

The majority of dogs with suspected allergic dermatitis in both groups did not have evidence of infection at their initial consultation or had superficial pyoderma. Skin conditions and infectious agents in the ears at initial consultations were investigated for any differences between oclacitinib treated dogs and dogs treated with other therapies. *Malassezia* infection was the most common agent present in ears in both groups, followed by cocci and then rods at their initial consultation. Besides the qualitative similarities at baseline, the regression analysis revealed that dogs treated with oclacitinib had significantly higher odds of presenting with superficial pyoderma and having *Malassezia* present in their pruritic ears. Since this study was conducted when oclacitinib was new in the Australian market, this could suggest that dogs receiving this drug were more chronic allergic cases and were not responding to other treatments, thus more likely to present with secondary infections.

Even though oclacitinib treated dogs seemed to have higher odds of presenting with infections, our descriptive analyses revealed that this group received fewer systemic and topical antimicrobials than dogs treated with other therapies. Further investigation using descriptive analysis suggested that there was a significant reduction in the use of systemic and topical antimicrobial therapies in the cases after initiation of oclacitinib usage. However, after adjusting for epidemiological factors such as signalment, drug use, skin condition and infectious agents in the ears, using regression analyses, there was no significant reduction in the overall use of systemic antimicrobials in oclacitinib treated dogs. Instead, these dogs were shown to have significantly lower odds of using specific systemic antimicrobials including cefovecin, amoxicillin plus clavulanic acid and topical chlorhexidine and neomycin. *Staphylococcus pseudintermedius* is often involved in canine skin infections and studies have shown that some strains have greater adherence to the corneocytes of inflamed skin of atopic dogs [13]. Oclacitinib targets key pathways to reduce inflammation which might reduce the capability of staphylococci to adhere to the skin and thus be involved in secondary infections, therefore reducing the need for antimicrobial therapies [1].

Our results indicate that oclacitinib treated cases were also more likely than controls to use fewer courses of topical and systemic glucocorticoids, antihistamines and medicated shampoos. The reduction in glucocorticoid use was also seen in our previous referral practice study, where it was suggested that veterinarians were probably inclined to avoid glucocorticoid use when treating with oclacitinib as there was little literature on the safety of using the two therapies simultaneously. This finding might also be linked to a more rapid alleviation of pruritis using oclacitinib and therefore reducing the need to prescribe other anti-pruritic therapies, although our data does not allow us to ascertain this hypothesis. Other studies have shown a faster reduction in pruritis when treated with oclacitinib compared to cyclosporine; however, a significant reduction in pruritis was only evident after 2 weeks of oclacitinib use compared to prednisolone [14, 15]. Interestingly, dogs treated with oclacitinib also had higher odds of being tested for allergies and to receive a hypoallergenic diet. This finding could partly be explained by the fact that oclacitinib cases were presented with more severe presentation at baseline compared to controls which may have triggered attending veterinarians to advise owners to carry out further testing and additional interventions. Another aspect could be due to the type of client who is willing to pay for regular oclacitinib treatments, which are expensive. These owners might be more willing to pay for more tests and to try allergic diets, although we do not have enough data to confirm this.

The findings of our study have a few limitations which are to be expected from a retrospective study using electronic patient records. First, there would be missing data from patients that did not return and was not re-examined at a participating clinic. Second, we cannot completely discard the potential for misclassification bias in our attempts to provide a definitive diagnosis for allergic dermatitis using the information available in the clinical records. While the examination notes were available for each dog, these contained a varying level of detail and completeness. As oclacitinib is indicated only in cases of allergic and atopic dermatitis, it is likely that cases consisted of a majority of atopic dogs; however, this may not be the case for controls. Thereby we attempted to minimise the impact of misclassification bias when prior to analysis we performed a misclassification assessment on a sample of the dataset using regular expression matching. As stated in the methods section, this misclassification was assessed at 11.3% which gave us confidence in our approach and the quality of the resulting dataset for analysis. We were also unable to differentiate between canine atopic dermatitis and flea allergy dermatitis due to the broad search terms used. The differences in allergic conditions might have affected the severity of skin conditions and hence the response to different treatments. The large size of the database would require more advanced in-text mining techniques to differentiate between specific allergic conditions, including natural language processing algorithms with some level of expert supervision. Third, we assumed that all medications flagged in the data set (Supplementary Table 3) were prescribed for the treatment of allergic dermatitis, including secondary infection. However, this is accounted for in classifying the examination notes, and these animals are included in the false positives. When interpreting our results it is important to keep in mind that we were not able to extract the dosage and administration routes of other antipruritic treatments, as this might affect how well pruritis is controlled by these treatments. And finally, our study is also prone to sampling bias since dogs included in the study were only from those general practice veterinary clinics contributing EPRs to the VetCompass Australia system. However, these practices are taken from across Australia and so are a much larger and more representative sample than that expected from referral dermatology practices or collected as a convenience sample from the general practice population.

The findings of this study have raised several areas of potential future research into oclacitinib to better understand its current use patterns and the biological mechanisms underlying its effects on clinical resolution. A few studies have found that oclacitinib is efficacious for the control of pruritus and atopic lesions [1, 10, 14, 16]. A geographical study would help determine whether location impacts the likelihood of an oclacitinib prescription, or whether certain types of clinics or particular veterinary companies are more likely than others to prescribe oclacitinib. Furthermore, a study that investigates the severity of atopic dermatitis and oclacitinib use, possibly using surveys for veterinarians or grading systems of the severity of skin lesions, may indicate whether oclacitinib is more likely to be prescribed in more severe cases of atopic disease.

## Conclusions

This case-control study showed that after adjustment for baseline skin or ear disease presentation oclacitinib reduced the subsequent number of courses of specific antimicrobials and other therapies in pruritic dogs with suspected allergic dermatitis. The information from this study may assist general practice veterinarians in planning for the long-term treatment of canine allergic dermatitis, with the desired effect of reducing antimicrobial use in these patients.

## Materials and methods

### Study population

The study dataset was collected by interrogation of the VetCompass Australia database, a national data collection system for veterinary medical records-based research (https://www.vetcompass.com.au). Bringing together all seven Australian veterinary schools, it is the only Australian wide companion animal surveillance system collating primary practice clinical records on diseases and treatments [17]. Electronic patient records (EPRs) were identified using a search in the free-text examination notes for commonly used terms for pruritic skin and terms focusing on allergy, with all terms used detailed in Supplementary Table 1. Further inclusion criteria were “canine” in the species field, an age of at least 1 year at consultation and consultation at a general practice participating in the VetCompass Australia programme from February 2008 to September 2018. The initial extracted dataset consisted of 65,597 unique dogs which presented to 80 veterinary clinics over 145,306 consultations. Further cleaning was conducted of the dataset using more detailed regular expression matches for terms representing pruritic dogs in the examination notes. Ultimately the final database included 40, 112 unique dogs after the removal of 39 under the age of one, and 25,446 who did not present for allergic skin disease (Supplementary Table 2). Data cleaning was performed using Stata version 15.1 (Stata Corporation, College Station, TX, USA).

### Case definition for case and control dogs

Cases were defined as pruritic dogs with presumptive diagnosis of allergic dermatitis who received oclacitinib, and controls were defined as pruritic dogs with suspected allergic dermatitis who received anti-pruritic treatments that were not oclacitinib. Our data suggested that it was not uncommon for cases (as well as controls) to received other antipruritic therapies along with oclacitinib which is displayed in more detail in Supplementary Fig. 1. The visit with oclacitinib in the billing field was flagged in the cases and the date for this was recorded. Courses of all treatments before or on that date were then summed for each patient as “before oclacitinib” courses, and all that occurred after this date were summed as “after oclacitinib” courses. Dogs were flagged if oclacitinib was billed in any consultation and these resulted in the population of cases (*n* = 1345). A case: control ratio of 1:4 was chosen, and 5380 controls were selected by simple random sampling of the non-flagged pool of dogs in the overall database.

### Data management

#### Data on medications

A table of medications of interest was generated (Supplementary Table 3). A regular expression match was used for each medication to flag the consultation with the medication present in the billing field, with the matches manually checked. After applying our regular expression algorithm, it was noted that most dogs did not have a live weight data available which hampered our ability to calculate the dosage of treatments. To circumvent this limitation we accounted for additional treatments in cases and controls by estimating the number of courses administered for a given therapy. One course of a particular treatment was considered to be each time a class of medication was flagged in the billing field; if a dog received both an injection and oral medication of the same class in the one consultation, such as an injection of dexamethasone then a course of prednisolone, then this was counted as one course. However, if an animal was given an application of one pharmaceutical and prescribed a different pharmaceutical, such as an injection of penicillin and then a course of cephalosporin, then this would count as two courses.

#### Data validation

The examination notes of 150 consultations randomly sampled from the dataset of both cases and controls were manually evaluated by a veterinarian to determine if the patient was likely to have allergic dermatitis. If the terms allergy, recurrent pruritis or atopy were included in the examination notes, the dog was deemed likely to have allergic dermatitis. The EPRs were excluded if the dog presented for reasons unrelated to atopy, such as dog fight wounds or surgery wound rechecks. The misclassification rate was found to be 11.3%.

### Statistical analyses

#### Descriptive analysis

The pipeline of statistical analyses was similar to our previous study [11] whereby an initial descriptive analysis was performed to compare a) topical and systemic antimicrobial use between cases (dogs treated with oclacitinib) and controls (dogs treated with other anti-pruritic) and b) within cases before and after the initial oclacitinib use. Wilcoxon rank sum tests were conducted with data from the first skin consultations for controls and with data from the first oclacitinib prescription for cases to determine whether topical and systemic antimicrobial courses differed significantly between cases and controls,. Wilcoxon signed-rank tests were performed to compare the differences within cases before and after oclacitinib.

#### Models adjusted for overall drug use and specific drug groups

Bernoulli logistic regression models were developed as described in the previous study [11], with oclacitinib treated dogs (cases) as the outcome of interest and dogs not treated with oclacitinib (controls) as the reference group. We extended the regression model of our previous study in that the different clinics (*N* = 77) were included as a random effect to account for multiple observations in the same clinical setting. Univariable logistic regression was initially used to identify predictor variables that would be included in the final multivariable models. Final multivariable models were achieved by using a manual backward stepwise variable selection process, and parameters with a *P*-value of ≤0.05 were termed significant. Two multivariable logistic regression models were developed; one focused on overall drug use Model (A) and the other focused on individual drug use (Model B).

#### Models adjusted for type of skin condition and presence of an ear condition

Model A and Model B were adjusted for evidence of concurrent skin infections and the presence of infectious agents in ears at baseline to determine if these conditions influenced antimicrobial use in the cases and controls. As described in the previous study [11], skin condition categories at the baseline consultation (first recorded skin allergy consultation for controls and first consultation of oclacitinib dispensation for cases) were allocated to three categories: allergic dermatitis without secondary infection, allergic dermatitis with secondary superficial bacterial pyoderma and allergic dermatitis with secondary deep bacterial pyoderma (Supplementary Table 5). The teram ear condition was used to include both infected and uninfected pruritic ears. The ear condition variable was divided into four categories. These included pruritic ears without the presence of an infectious agent, presence of cocci, presence of rods, and presence of *Malassezia*. All statistical analyses were performed using Stata version 15.1 (Stata Corporation, College Station, TX, USA) and Microsoft Excel.

## Supplementary Information

**Additional file 1.**

## Data Availability

The datasets used and analysed during the current study are available from the corresponding author on reasonable request.
