# Supplementary material for: The effects of oclacitinib treatment on antimicrobial usage in allergic dogs in primary practice: an Australia wide case-control study
Source: BMC Vet Res. 2022 Apr 27;18:151. doi: 10.1186/s12917-022-03255-y (PMC9044603; doi:10.1186/s12917-022-03255-y)
Supplement: Supplementary file 1 — Additional file 1. [file 12917_2022_3255_MOESM1_ESM.docx]

**Supplementary File**

**Supplementary Table 1.** Key words and phrases generated for VetCompass™ data extraction

| **Keywords** |
| --- |
| pruritus  itching  dermatitis  atopy  atopic  atopic flare  atopic dermatitis  allerg  allergy  allergic dermatitis  dermat  allerg test  dermat  flea  allerg  flea  dermat  food allerg dermat  food allerg  diet trial  pyoderma AND sarcoptic mange  chronic inflammatory skin disease AND acute moist dermat  hot spot AND yeast dermatitis  flea allerg dermat AND allerg  dermat AND allerg  immunotherapy AND allerg  test AND atopic  dermat AND flea  allerg AND flea  dermat AND food  cutaneous AND food  dermat AND food |

**Supplementary Table 2.** Terms used in the regular expression matches in consultation examination notes

| **Keywords in consultations used to remove dogs** |
| --- |
| not atopy\| not atopic\| not pruritic  no allergies\| not allergies\| no known allergies\| no known allergy  not allergic\| not allergy\| no pruritis  not atopy\| not atopic\| not pruritic  lump removal\|bandage\|bandages\|elastoplast\|solosite\|melolin\|ringworm\|ringworms  stitches\|s/o\|sutures\|diarrhoea\|gi\|git\|vomit\|diarrhoea\|tumour\|tumor\|lump  haematoma\| mass removal\| rebandage\| ultrasound\| lumpectomy  torn nail\| surgery\| wound\| dental  cruciate\| suture\| coughing\| sneezing\| bronchitis\| dog fight\| dog attack  neoplasia\| vomit\| upper airway\| upper air way\| mass  injured\| castrate\| spey\| spay  limping\| lame\| limp\| tick\| allergic asthma\| allergic bronchitis\| allergic conjunctivitis  anal glands\| anal gland\| anus\| radiographs  uti\| urinary\| stranguria\| haematuria\| lameness\| hypothyroid\| hypot  lip fold dermatitis\| allergic rhinitis\| dectomax\| splint\| vulval dermatitis  vulva fold dermatitis\| vulval fold dermatitis\| dermatitis of vulva  heart failure\| dry eye\| vaccine reaction |
| **Keywords in consultations used to keep dogs** |
| rash\| itch\| itchy\| itching\| scratch\| scratching\| hypoallergenic\| hypo allergenic\| allergen\| licks feet\| skin lesions  otitis\| dermatitis\| hot spot\| hotspot\| hot-spot\| skin check\| eczema\| skin condition\| ear issues  ear check\| check skin\| skin irritation\| alopecia\| erythema\| chewing feet\| lick at feet  flared\|scratchign\|scurf\|scurfy\|pruritic\|pododermatitis\|erythematous\|chews feet  skin referral\| ear infection\| skin infection\| pyoderma\| ex skin\| examine skin  skin appointment\| skin irritated\| skin irritation\| prurutic\| ch skin\| skin problem\| recheck skin  skin specialist\| dermatologist\| dermatology\| red skin\| pruritus\| hair loss\| painful skin\| skin problems  skin issues\| mucky ears\| sore ears\| ear pain\| painful ears\| painful ear  ears: inflammed\| ears: inflammation\| ears: chronic inflammation\| ears: infected\| skinology  recheck ear\| ear infections\| flare\| atopy\| check ears\| yeast\| yeasty\| malassezia\| sensitive skin\| apoquel\| allergy |

**Supplementary Table 3.** Treatments flagged, identified and classified in cases and controls

| **Pharmaceutical group** | **Class** | **Search terms** |
| --- | --- | --- |
| Antibiotics | Tetracycline | Doxycycline, tetracycline  oxycycline |
|  | Cephalosporin first generation | Cephalexin, cephaforte |
|  | Cephalosporin third generation | Cefovecin |
|  | Penicillin without beta-lactamase inhibitors | Amoxicillin, amoxicillin, ampicillin, benacillin, penicillin, benzylpenicillin, depocillin, propercillin |
|  | Penicillin with beta-lactamase inhibitors | Noroclav, amoxyclav, clavulox, amoxycillin clavulanic acid, ticarcillin clavulanate, timentin |
|  | Sulfonamide | Trimethoprim, sulfadiazine |
|  | Nitroimidazole | Metronidazole |
|  | Lincosamide | Clindamycin, antirobe |
|  | Fluoroquinolone | Baytril, enroflaxacin, cirofloxacin |
| Antifungals |  | Ketoconazole, itraconazole  fluconazole, terbinafine  voriconazole, posaconazole |
| Cyclosporine |  | Cyclosporine, atopica, neural |
| Corticosteroids |  | Prednisolone, prednisone, delta cortef, methylprednisolone, cortisone, dermapred, macrolone, niralone, preddy granules, predmix, prednil, pred-x, solone, dexamethasone, triamcinolone, delta albaplex tabs, apex pmp, dermotic, hydrocortisone, neocort, neotopic, cortavance, canaural, betamethasone |
| Antihistamines |  | Niralone (contains c/s), chlorpheniramine, dexchlorpheniramine, cetirizine, hydroxyzine, cyproheptadine, terfenadine, clemastine, trimeprazine, fexofenadine, niramine, diphenhydramine |
| Topical antimicrobials (these medications contain more than one type of medication) |  | Prednoderm, dermapred, dermotic, isaderm, fuciderm, neocort, cortavance, neotopic-h, panolog, apex antibiotic, surolan, dermocil, dermaclens, dermoscent bio balm, paw triderm, otoflush, otiderm, otomax, canaural, aurizon, baytril ear drops, ciproxin hc ear drops |
| Omega 3/6 fatty acids |  | Omega, omegaderm, omega-3, megaderm, dermega |
| Hypoallergenic diets |  | z/d, d/d, advance dog sens skin, delicate care dog skin or stomach, euk dog sensitive skin, royal canin dog skin support, hills dog sens skin, nb delicate care dog skin, rcw dental and skin adult, royal canin canine skin support, euk dog dermatosis, hills derm defence, ppvd dry ha canine hypoallergenic |
| Medicated shampoos |  | Dermcare ear culture, fidos everyday shampoo, fidos white & bright conditioner, paw 2 in 1 conditioner & shampoo, paw 2in1 cond shampoo, paw classic care shampoo, natural shampoo 250ml dermcare, natural shampoo 500ml, paw nutriderm replenishing shampoo, paw puppy shampoo, troy pet gloss conditioner, wash mate shampoo, otoflush |
| Flea treatments |  | Advantage, advantix, advocate, bravecto, capstar, comfortis, nexgard, panoramis, revolution, sentinel, simparica, imidacloprid, permethrin, moxidectin, fluralaner, nitenpyram, spinosad, afoxolaner, selamectin, milbemycin oxime, sarolaner |
| Thyroid medication |  | Oroxine, thyroxine |
| Allergy vaccinations (immunotherapy) |  | Recorded as 1 for vaccinated and 0 for unvaccinated as allergens were not specified in item name |
| Individual drugs | Hydrocortisone | Hydrocortisone |
|  | Chlorpheniramine | Chlorpheniramine |
|  | Chlorhexidine | Chlorhexidine, chlorhex, malaseb, pyohex, topizole |
|  | Neomycin | Neomycin, neocort, neotopic |
|  | Polymixin B | polymixin B, apex pmp, dermotic |
|  | Amoxycillin clavulanic acid | Noroclav, amoxyclav, clavulox, amoxycillin clavulanic acid |

**Supplementary Table 4.** The percentage distribution of different skin and ear condition categories in cases and controls at baseline.

| **Skin condition categories** | **Control (%)** | **Case (%)** |
| --- | --- | --- |
|  |  |  |
| Allergic dermatitis without infection | 92 | 93 |
| Unknown | 6 | 4 |
| Superficial pyoderma | 3 | 2 |
| Deep pyoderma | 0 | 0 |
| **Ear condition categories** | **Control (%)** | **Case (%)** |
|  |  |  |
| Unknown/not an ear condition | 89 | 88 |
| Cocci present | 3 | 2 |
| Rods present | 1 | 0 |
| Malassezia present | 8 | 9 |

**Supplementary Table 5. Search terms used for skin condition categories**

| **Skin condition categories** | **Search terms** |
| --- | --- |
| Superficial pyoderma | 1. Papules, pustules, erythema, collarettes 2. Cytological evidence of infections 3. Antibiotic or intensive topical therapy dispensed 4. Clinical diagnosis 5. At least 3 of the above should be identified |
| Deep bacterial pyoderma | 1. Nodules, draining tracts, cellulitis etc (furunculosis is technically a histopathological diagnosis but commonly used as a descriptor in records). 2. Clinical diagnosis 3. Antibiotics dispensed for more than 8 weeks |
| Allergic dermatitis with no infection | 1. No systemic or topical antibiotics dispensed 2. No clinical mentioned of pyoderma |


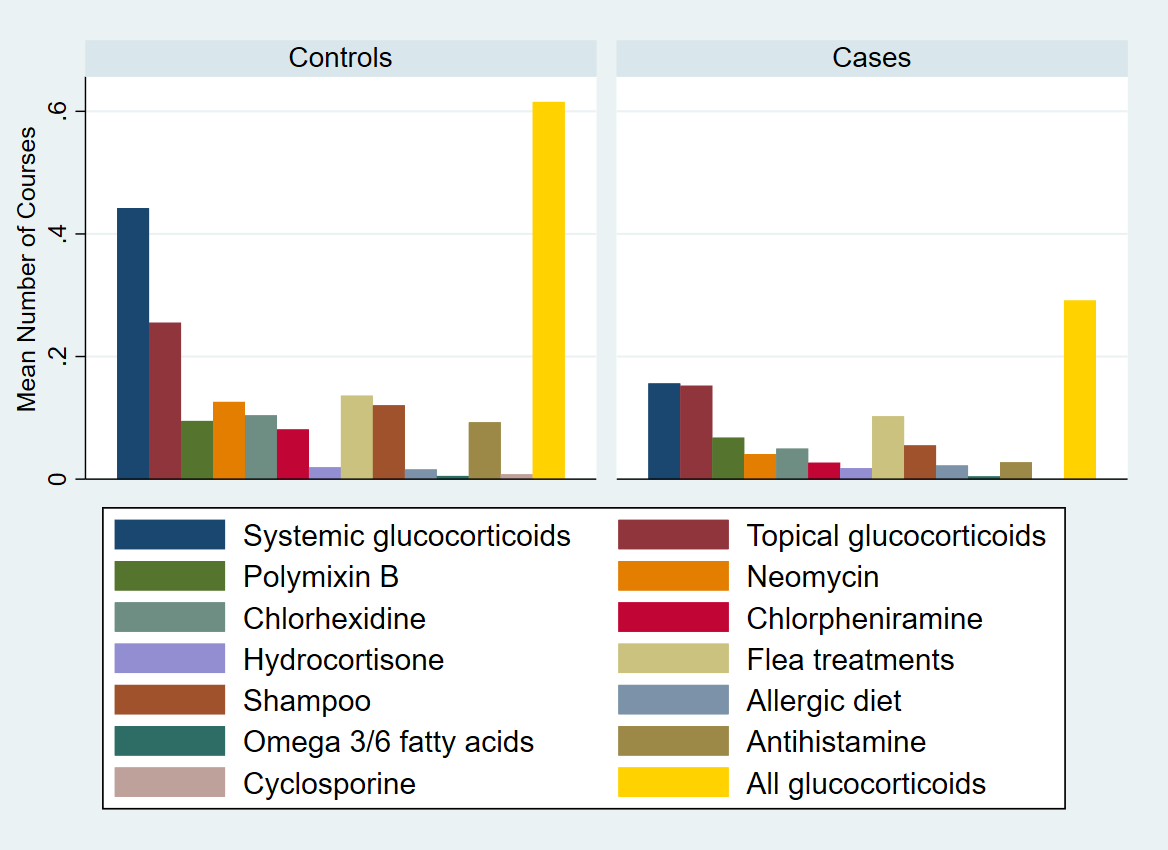


Supplementary Figure 1. Bar charts displaying the mean number of courses of antipruritic treatments other than oclacitinib used by 5380 controls (after their initial skin consultation) and 1345 cases (after initial oclacitinib use).
